# Low bone density, vertebral fracture and FRAX score in kidney transplant recipients: A cross-sectional cohort study

**DOI:** 10.1371/journal.pone.0251035

**Published:** 2021-04-30

**Authors:** Arzu Velioglu, Burcu Kaya, Basar Aykent, Bige Ozkan, Melis Sevil Karapinar, Hakki Arikan, Ebru Asicioglu, Onur Bugdaycı, Dilek Gogas Yavuz, Serhan Tuglular

**Affiliations:** 1 Division of Nephrology, Department of Internal Medicine, Marmara University School of Medicine, Istanbul, Turkey; 2 Department of Radiology, Marmara University School of Medicine, Istanbul, Turkey; 3 Division of Endocrinology, Department of Internal Medicine, Marmara University School of Medicine, Istanbul, Turkey; University of Toledo, UNITED STATES

## Abstract

**Background:**

Kidney transplantation (KT) recipients are at increased risk of low bone density (LBD) and fractures. In this retrospective study, we investigated bone mineral density (BMD), vertebral fractures, calculated risk for major osteoporotic fractures (MOF), and hip fractures in the KT recipients.

**Patients-method:**

Patients who completed at least one year after KT were included in the analysis. Demographic, clinical, and laboratory data were recorded. Measurements of BMD were performed by dual-energy X-ray absorptiometry. Vertebral fractures were assessed using semi-quantitative criteria with conventional radiography. The ten-year risk for MOF and hip fracture were calculated using the FRAX@ tool with BMD.

**Results:**

One hundred fifty-three KT recipients were included in the study. The population included 77 women. The mean age at evaluation was 46,5±11,9 years. Seventy-eight (50.9%) patients had normal femoral neck BMD while osteoporosis and osteopenia at the femoral neck were present in 12 (7.8%) and 63 (41.1%) of the patients, respectively. Age at evaluation was the risk factor for LBD (OR 1.057; 95% CI 1.024–1.091; p = 0.001). In female KT recipients, LBD was principally affected by menopausal status whereas in males, mammalian target of rapamycin (mTOR) inhibitor use and lower BMI levels were the risk factors. The prevalent vertebral fracture was found in 43.4% of patients. In multivariate analysis, only steroid use (OR 0.121; 95% CI 0.015–0.988; p = 0.049) was found to be associated with prevalent fracture. Among all KT recipients, 1.9% had a high MOF probability (≥20% risk of fracture), and 23.5% had high hip fracture probability (≥3% risk of hip fracture) according to FRAX.

**Conclusion:**

Exploring the prevalence of LBD and vertebral fracture and the risk factors would help clinicians to modify long-term follow-up strategies. Furthermore, the high hip fracture risk probability in our cohort suggested that there is a need for longitudinal studies to confirm the validity of the FRAX tool in the transplant population.

## Introduction

Mineral and bone disease in kidney transplant patients is a major cause of morbidity and mortality in early and late post-transplant period. Kidney Disease: Improving Global Outcomes (KDIGO) guidelines published in 2017 with a special section on the management of CKD-MBD in transplant patients recommends Bone Mineral Density (BMD) evaluation to predict fracture risk in kidney transplant recipients especially in the first three months after transplantation [1]. This evaluation is especially important to detect patients eligible for treatment for osteoporosis with a perspective to prevent fractures. For the general population, The National Osteoporosis Foundation recommends any of the following criteria to be used to determine eligibility for treatment: T-scores less than or equal to -2.5 at the femoral neck, total hip, or lumbar spine; previous hip or vertebral fractures; and 10-year major osteoporotic fracture (MOF) probability greater than or equal to 20% or 10-year hip fracture probability greater than or equal to 3% [2]. Although BMD assessments and its value in transplant recipients are well known, the importance of screening for vertebral fractures remains mostly unrecognized in transplant practice. The presence of osteoporosis increases the risk of fractures in the general population and the most common osteoporotic fractures seen are in vertebral bones. Osteoporotic vertebral fractures not only demonstrate reduced bone strength but also increase the risk of new fractures and death [3]. The prevalence of vertebral fractures in kidney transplant recipients seems to be largely variable with reported incidence ranging between 15% to 66.0%, depending on the diagnostic criteria used [4–8]. Data on association of decreased BMD with fracture prevalence in transplant recipients are limited and the value of assessment for BMD and vertebral fracture beyond 12 months after kidney transplantation remains undetermined [1]. Furthermore, fracture risk assessment with The Fracture Risk Assessment Tool (FRAX®) for 10-yr risk of hip and MOF has not been validated in transplant recipients and there is need for clinical studies to determine the usefulness of these tools.

Although the general risk factors for low bone density and vertebral fractures are well documented in transplant recipients, their prevalence and transplant specific risk factors may vary. Low BMD and vertebral fractures are more commonly reported in female gender but, there are also many studies showing that, the prevalence of bone metabolism disorders is not at all negligible in men [8–10].

With the following study, we aimed to investigate the prevalence of and risk factors for vertebral fracture and osteoporosis/osteopenia in our kidney transplantation recipients using the FRAX score, to explore its usefulness in transplant population.

## Patients and methods

Kidney transplant recipients who were evaluated for bone metabolism at Marmara University School of Medicine Transplant clinic between January 2017 and December 2018 were included in the study.

Patients with stable graft function who remained on the same immunosuppressive treatment for at least 6 months were analyzed. Patients with transplantation time less then 12 months and patients already on bisphosphonate or denasumab treatment were excluded from analysis. Patient demographics and clinical risk factors including age, sex, body mass index (BMI), comorbid conditions (diabetes, hypertension, coronary artery disease), current smoking status, cause of primary renal disease, pre-transplant dialysis type and dialysis vintage, menopausal status for women, current medications, family and personal history of bone fractures, were obtained from patients’ file and were also confirmed with face to face interrogation. All data were retrospectively collected from patients’ files and electronic archive system in 2019. Written informed consents were taken at the clinical interview scheduled for face-to-face interrogation in 2019.

### Biochemical parameters

Biochemical analyses including serum creatinine, calcium, phosphate, 25- hydroxyl vitamin D, intact parathormone, total alkaline phosphatase as well as osteocalcine and serum c-telopeptide (CTX) were measured as indicators of bone metabolism. Osteocalcine was measured by radioimmunoassay (Cis-Bio International, Gif-sur-Yvette) and serum CTX was measured by immuno-enzyme assay (ELISA). Blood samples were obtained after overnight fasting. Estimated glomerular filtration rate (eGFR) was calculated for each patient by Modification of Diet in Renal Disease (MDRD) equation. All laboratory results were obtained from electronic archive system.

### Bone mineral density measurement

BMD measurements were performed by DEXA (GE Lunar Corp, Madison, WI, USA) at the hip, lumbar spine L1-L4, and femoral neck. We assessed the areal BMD (g/cm2) and T-Scores using the National Health and Nutrition Examination Survey reference population. We used the WHO definitions of BMD categories: normal bone density (T-Score ≥ –1.0), osteopenia (T-Score between −1.0 and −2.5), and osteoporosis (T-Score ≤ –2.5) to assess BMD. Patients with osteopenia or osteoporosis were defined as patients with low bone density (LBD).

### Vertebral fracture assessment

Lateral chest and lumbar radiographs were used for the diagnosis of vertebral fractures with a semi-quantitative method defined by Genant and Jergas [11]. Vertebrae were graded on visual inspection as normal (grade 0), mildly deformed (grade 1, approximately 20–25% reduction in anterior, middle, and/or posterior height and a 10–20% reduction in area), moderately deformed (grade 2, approximately 25–40% reduction in any height and 20–40% reduction in area), and severely deformed (grade 3, approximately 40% reduction in any height and area). Only prevalent vertebral fractures defined as Genant grade 2 or higher were included the statistical analysis [12, 13]. All radiographs were read by an experienced endocrinologist (DGY), re-read in consensus readings and verified by an expert radiologist (OB). On average, we evaluated 8,1±4,05 range [4–17] vertebrae per patient.

### Fracture risk assessment

Fracture risk was calculated by The World Health Organization’s Fracture Risk Assessment Tool (FRAX) based on patient’s age, sex, and clinical risk factors. Ten-year probability of a Major osteoporotic fracture (MOF) and hip fracture was calculated based on the Turkish FRAX tool (FRAX Web, version 4.1) [14]. Calculated MOF risk over %20 and hip fracture risk over %3 were classified as high risk. FRAX predictions agree closely with the observed fracture rates in the general Turkish population [15].

This study complied with the principles of the Declaration of Helsinki and was approved by Institutional Research Ethics Board of Marmara University School of Medicine (approval ID: 09.2018.432).

#### Statistical analysis

The characteristics of the study patients were expressed as mean or median, as appropriate for categorical variables, percentages and variables with continuity. Mann-Whitney U-test and Kruskal-Wallis test were used for comparing median variables between groups and independent samples *t*-test and one-way ANOVA tests were used for comparing parametric variables. For comparing categorical data, Chi-square and Fisher’s exact test was used. A Binary multivariable logistic regression analysis was used to investigate the risk factors for the development of LBD and/or prevalent vertebral fracture. Covariates used in multivariate model were determined according to univariate analysis. Additionally, steroid use was also included in the multivariate analysis since it is a well-known strong risk factor for LBD. For all statistical analysis, *p* value <0.05 was considered significant. All data are analyzed with SPSS (version 20.0; SPSS Inc, Chicago, IL) statistical package.

## Results

A total of 162 transplant recipients underwent bone metabolism evaluation during the study period. Nine patients were excluded from the study since seven were already under treatment of bisphosphonates or denasumab and two were not eligible for vertebral facture assessment due to severe kyphoscoliosis. Finally, the cohort included 153 patients whose demographic and clinical characteristics are summarized in Table 1.

**Table 1:** Demographic and clinical parameters of the study population.

Parameters	Total (n = 153)	Female (n = 77)	Male (n = 76)	*p* value
Age (years)				
At the time of transplantation	39,1±12,1	45,3±12	47,6 ±11,7	0.227
At the time of bone assessment	46,5±11,9	38,4±13,1	39,8±11,1	0.482
BMI (kg/m^2^)	25,9 ± 4,8	25,5±5,1	26,4±4,4	0.282
BMI groups				0.170
Normal	68 (44.4%)	40 (51.9%)	28 (36.8)	
Overweight	59 (38.6%)	24 (31.2%)	35 (46%)	
Obese	26 (17%)	13 (16.9%)	13 (17.1%)	
Duration of dialysis (months) median [range]	35 [2–216]	36 [3–216]	28 [2–168]	0.204
Post-transplant time (months) median [range]	86,4 [12,1–233,2]	86,8 [12,1–233,2]	85,4 [17,4–225,3]	0.240
Post-transplant time groups				0.059
1–3 years	25 (16.3%)	18 (23.4%)	7 (9.2%)	
3–5 years	25 (16.3%)	11 (14.3%)	14 (18.4%)	
>5 years	103 (67.3%)	48 (62.3%)	55 (72.4%)	
Rejection (n,%)	43 (28.1%)	18 (23.4%)	25 (32.9%)	0.212
Comorbid conditions (n,%)				
Hypertension	121 (79.1%)	54 (70.1%)	67 (88.2%)	0.009
DM or PTDM	51 (33.3%)	28 (36.4%)	23 (30.3%)	0.494
CAD	18 (11.8)	6 (7.8%)	12 (15.8%)	0.126
IS treatments (n,%)				
Tacrolimus/Cyclosporine	130 (84.9%) / 11(7.2%)	71(92.2%)/4(5.2%)	59(77.6%)/7(9.2%)	0.237
mTOR inhibitors	33 (21.6%)	16 (20.8%)	17(22.4%)	0.846
MMF/MPA	122 (79.7%)	58 (75.3%)	64 (84.2%)	0.228
AZA	10 (6.5%)	8 (10.4%)	2 (2.6%)	0.098
Steroid use (n,%)	133 (86.9%)	69 (89.6%)	12 (84.2)	0.348

BMI, Body Mass Index; DM, Diabetes Mellitus; PTDM, Post Transplantation Diabetes Mellitus; CAD, Coronary Artery Disease; IS, Immunosuppressive.

Briefly our cohort consisted of mostly living kidney recipients (75.8%; n = 116) and the median time since kidney transplantation was 86,4 months; range [12,1–233]. The most common primary kidney diseases were as follows: Glomerulonephritis in 22.2%, hypertensive nephropathy in 8.5%, polycystic kidney disease in 7.1% and diabetes mellitus in 6.5% of the patients. About 43% of the female patients were in the post-menopausal period. The average BMI was 25.9 kg/m^2^ while 55.6% of patients were overweight or obese. Almost 87% of the patients were on maintenance steroid treatment while only 20 (13.1%) patients were on a steroid-free regimen. History of parathyroidectomy was present in six patients at the time of evaluation. History of any fracture was reported in nine cases. Nutritional vitamin D and calcium supplements were used in 20.3% and 28.1% of patients, respectively. At the time of the study, nine of the patients were on calcitriol while one patient was on cinacalcet. Male and female recipients had similar characteristics, except hypertension, which was more prevalent in male recipients compared to females (Table 1).

### Bone mineral density

At the time of evaluation 78 (50.9%) patients had normal femoral neck BMD while osteoporosis and osteopenia at femoral neck were present in 12 (7.8%) and 63 (41.1%) of the patients, respectively. Osteoporosis and osteopenia prevalence were 7.2% and 28.1% at the lumbar spine and 7.7% and 37.9% at the total hip, respectively. Therefore, LBD was more frequent in femoral neck than in other sites. Patients with LBD were significantly older than patients with normal BMD (50±12,1 vs. 43±10,6 years; p<0.001).

There were no differences among patients with normal BMD and those with LBD in terms of dialysis vintage, primary kidney disease, BMI at evaluation and co-morbidities. Regarding biochemical parameters, we found that alkaline phosphatase levels were higher in LBD group when compared to patients with normal BMD (95,5±31,2 U/L vs. 80,5±27,1 U/L; p = 0.003). Only the eGFR values in osteoporosis group were lower than the other groups (p = 0.040). There were no differences in the number of patients receiving tacrolimus, cyclosporine and steroid. However LBD was more frequent in patients on mammalian target of rapamycin (mTOR) inhibitor treatment (69.7% vs. 43.3%, p = 0.010). Comparisons of the patient groups are shown in Table 2. In multivariate analysis, age at evaluation (OR 1.049; 95% CI 1.015–1.084; p = 0.004) and serum alkaline phosphatase levels (OR 1.017; 95% CI 1.003–1.030; p = 0.015) were found as risk factors for LBD. However steroid use (OR 0.641; 95% CI 0.201–2.047; p = 0.453) and mTOR inhibitor use (OR 0.404; 95% CI 0.158–1.030; p = 0.058) were not found as significant risk factors for LBD in whole group.

**Table 2:** Comparison of the patients’ demographic and clinical parameters regarding BMD status.

	Normal BMD (n = 78)	Low Bone Density (n = 75)		*p* value
		Osteopenia (n = 63)	Osteoporosis (n = 12)	
Age at evaluation (years)	43±10,6	49,5±12#	52,7±13,2*	**0.001**
Age at transplantation (years)	36±10,6	42±12,6#	43,7±14,6*	**0.005**
Sex (Female/Male) (n,%)	42(53.8%)/36 (46.1%)	28(44.4%)/35(55.5%)	7(58.3%)/5(41.7%)	0.457
Menopausal status (n,%)	9 (21.4%)	18 (64.3%)	6 (85.7%)	**<0.001**
Duration of dialysis (months) (median)	24	43	30	0.313
Time to evaluation from transplantation (months) (median)	86,9	82,1	89,7	0.414
BMI (kg/m^2^)	26,5±4,8	25,4±4,8	25±4,9	0.288
BMI groups (n,%)				0.555
Normal	30 (38.4%)	31(49.2%)	7 (58.3%)	
Overweight	32 (41%)	24 (38.1%)	3 (25%)	
Obese	16 (20.5%)	8 (12.7%)	2 (16.6%)	
DM or PTDM (n, %)	26 (33.3%)	21 (33.3%)	4 (33.3%)	1.000
Creatinine (mg/dL)	1,2±0,5	1,37±0,83	1,5±0,72	0.186
eGFR (ml/min)	69,7±25,1	63,3±23,2	54±25,8*	0.069
Calcium (mg/dL)	9,7±0,62	9,8±0,67	9,6±0,67	0.465
Phosphate (mg/dL)	3,18±0,62	3,21±0,7	3,36±0,78	0.693
PTH (pg/mL)	69,8	86,8#	64,95	0.265
(median)				
PTH groups (n,%)				0.111
<70	40 (51.3%)	25 (39.7%)	7 (58.3%)	
70–150	32 (41%)	26 (41.3%)	2 (16.7%)	
>150	6 (7.7%)	12 (19%)	3 (25%)	
25-OH vitamin D (ng/mL)	20,7 ±8,8	23,5±11	26,3±10,3	0.115
Vitamin D status (n,%)				0.628
Normal	27 (34.6%)	15 (23.8%)	3 (25%)	
Insufficiency	38 (48.7%)	32 (50.8%)	6 (50%)	
Deficiency	12 (15.4%)	14 (22.2%)	3 (25%)	
Alkaline Phosphatase (U/L)	80,6±27,1	95,15±32,6#	97,27±23,6	**0.013**
Osteocalcine (μg/L)	7,6±5,5	7,9±6,1	9,8±7,4	0.562
c-telopeptide (μg/L)	0,59±0,43	0,59±0,34	0,73±0,47	0.766
CNI (n,%)				0.098
Tacrolimus (yes)	72 (92.3%)	49 (77.8%)	9 (75%)	
Cyclosporine (yes)	4 (5.1%)	6 (9.5%)	1 (8.3%)	
mTOR inhibitors (yes) (n,%)	10 (12.8%)	18 (28.6%)	5 (41.7%)	**0.016**
Steroid use (n,%)	65 (83.3%)	56 (88.9%)	12 (100%)	0.234
Rejection (n,%)	22 (28.2%)	18 (28.6%)	3 (25%)	0.968

BMI, Body Mass Index; DM, Diabetes Mellitus; PTDM, Post Transplantation Diabetes Mellitus; CNI, Calcineurin Inhibitor; mTOR, mammalian target of rapamycin; eGFR, estimated glomerular filtration rate.

#p<0.05 Normal BMD vs. Osteopenia.

*p<0.05 Normal BMD vs. Osteoporosis.

Although there was no significant difference between genders, except for the presence of hypertension in men, post-menopausal women tended to have a higher prevalence of osteopenia and osteoporosis. The comparison of significant study parameters in patients with and without low bone density with regard to gender is summarized in Table 3. In women, menopausal status was the major risk factor for low BMD, whereas in men, mTOR inhibitor use and lower BMI were the major risk factors.

**Table 3:** Comparison of statistically significant risk factors in patients with and without low bone density with regards to sex.

Risk Factors	*Univariate Analysis*			*Multivariate analysis*	
	Low Bone Density (-)	Low Bone Density (+)	*p value*	*OR (%95CI)*	*p value*
***Female Recipients***	n = 42	n = 35			
Age at transplantation (years)	34±11	43,7±13,5	**0.001**	0.985 [0.917–1.058]	0.675
Menopausal status (n,%) (yes)	9 (21.4%)	24 (68.6%)	**<0.001**	11.203 [1.65–75.82]	**0.013**
Steroid use (n,%)	37 (88.1%)	32 (91.4%)	**0.721**	1.409 [0.25–7.82]	**0.695**
mTOR inhibitor use (n)	6 (14.3%)	10 (28.6%)	**0.162**	-	**-**
BMI (kg/m^2^)	25.5±5.2	25.6±5.2	**0.953**	-	**-**
***Male Recipients (n = 76)***	n = 36	n = 40			
Age at transplantation (years)	38.5±9.8	41±12.2	**0.322**	-	**-**
Steroid use	28 (77.8%)	36 (90%)	**0.209**	2.994 [0.688–13.027]	**0.144**
mTOR inhibitor use (n, %)	4 (11.1%)	13 (32.5%)	**0.030**	4.406 [1.177–16.498]	**0.028**
BMI (kg/m^2^)	27,8±4.04	25,1±4,4	**0.008**	0.851 [0.747–0.970]	**0.016**

mTOR, mammalian target of rapamycin; BMI, Body mass index.

### Vertebral fracture

A total of 129 patients had imaging studies done for vertebral fracture. Ninety-eight of them (64.1%) had at least grade 1 vertebral fracture. Prevalent fracture was found in 43.4% (n = 56) of all patients. All the patients with radiologically confirmed vertebral fractures were asymptomatic. The clinical, laboratory parameters and DEXA results of the patients according to the presence of prevalent fracture are shown in Table 4. The significantly different clinical factors between the patients with and without prevalent vertebral fractures were being diabetic and steroid use (p = 0.039, p = 0.007, respectively). Patients who had diabetes mellitus starting from before the kidney transplantation or who developed post-transplant diabetes mellitus (PTDM) had a significantly higher rate of prevalent fractures. Patients with steroid free regimen tended to have less vertebral fracture. As expected, BMD and T-score at lumbar spine was found to be significantly lower in patients with vertebral fracture than those without a vertebral fracture (p = 0.023 and p = 0.018). In multivariate analysis, only steroid use (OR 0.121; 95% CI 0.015–0.988; p = 0.049) was found to be associated with prevalent fracture whereas lumbar spine T-score was marginally statistically significant (OR 0.744; 95% CI 0.553–1.002; p = 0.051). When we analyzed the risk factors for prevalent vertebral fracture differentially we did not find a gender specific risk factor for either sex.

**Table 4:** Characteristics of the patients and vertebral fractures.

Parameters	Prevalent fracture (+) (n = 56)	Prevalent fracture (-) (n = 73)	*p* value
Age at evaluation (years)	48,8±11,9	45,6±12,4	0.153
Age at transplantation (years)	41,7±11,9	38,5±12,5	0.135
Sex (Female/Male)(n,%)	33(58.9%)/28(50%)	35(47.9%)/38(52%)	0.286
Menopausal status (n,%)	16(48.5%)	15(42.8%)	0.808
Primary kidney disease (n,%)			
Glomerulonephritis	9(16.1%)	19(26%)	0.242
Dialysis vintage (months)	85±46,8	87,3±50.7	0.791
Time to evaluation from transplantation (months) (median)	76.35	79.5	0.907
BMI (kg/m^2^)	25,6±4,6	25,8±5,15	0.720
BMI groups (n,%)			0.662
Normal	25(76.7%)	35 (47.9%)	
Overweight	23(41.1%)	25(34.2%)	
Obese	8 (14.3%)	13(17.8%)	
DM or PTDM (n,%)	25(76.7%)	19(26.2%)	**0.039**
Creatinine (mg/dL)	1,18±0,53	1,29±0,54	0.263
eGFR (ml/min)	69,6±25,3	65,3±24,5	0.333
Calcium (mg/dL)	9,8±0,7	9,8±0,63	0.944
Phosphate (mg/dL)	3,25±0,5	3,18±0,7	0.511
PTH (pg/mL) (median)	84.45	67	0.285
PTH groups (n,%)			0.175
<70	21 (37.5%)	39(53.4%)	
70–150	26 (46.4%)	27 (37%)	
>150	9 (16.1%)	7 (9.6%)	
25-OH vitamin D (ng/mL)	23,2±10,9	22,6±10,4	0.742
Vitamin D status (n,%)			0.184
Normal	12 (21.4%)	23 (31.5%)	
Insufficiency	34 (60.7%)	32 (43.8%)	
Deficiency	10 (17.8%)	17 (23.3%)	
Alkaline Phosphatase (U/L)	92,2±30	84,2±31,5	0.158
Osteocalcine (μg/L)	7,6±5,4	8,4±6,3	0.447
c-telopeptide (μg/L)	0,59±0,36	0,60±0,41	0.878
CNI (n,%)			0.463
Tacrolimus (yes)	46 (82.1%)	65 (89%)	
Cyclosporine (yes)	5 (8.9%)	5 (6.8%)	
mTOR inhibitors (yes) (n,%)	15 (26.8%)	11(15.1%)	0.123
Steroid use (n,%)	54 (96.4%)	59 (80.8%)	**0.007**
Rejection (n,%)	14 (25%)	20 (27.4%)	0.841
L1-L4 (g/cm^2^)	1,052±0,17	1,12±0,16	**0.023**
L1-L4 T score	-0.717±1,41	-0.110±1,35	**0.018**
Total hip (g/cm^2^)	0,865±0.126	0,906±0,149	0.101
Total hip T score	-1,157±0,991	-0.775±1,121	0.064
Femoral neck (g/cm^2^)	0,829±0,119	0,873±0,158	0.085
Femoral neck T score	-1,235±0,948	-0,943±1,258	0.158

### Fracture risk assessment

Median 10-year probability of a MOF and hip fracture calculated using FRAX tool were 7.0%, range [2.1%-48%] and 1.3%, range [0%-43%] respectively. Among all KT recipients, 1.9% (n = 3;) had a high MOF probability (≥20% risk of fracture), and 23.5% (n = 36) had high hip fracture probability (≥3% risk of hip fracture). We did not find a statistically significant difference in FRAX scores with respect to genders.

## Discussion

In this retrospective observational study, we evaluated prevalence and risk factors of low bone density and vertebral fracture in kidney transplant recipients who had completed at least 12 months follow up after transplantation The prevalence of low bone density was 49%in our cohort which is comparable to previous studies [4, 5, 16]. Although the reported age at time of transplantation and at the time of evaluation, mTOR inhibitor use and alkaline phosphatase levels between the patients with or without low bone density, were significantly different, we found that independent risk factors for low bone density were age at the time of evaluation and higher alkaline phosphatase levels.

In the general population, there is significant reduction in bone formation with increasing age. This is mostly due to a shift from osteoblastogenesis to predominant adipogenesis in the bone marrow, which in turn affects matrix formation and mineralization [17]. The decrease in BMD starts after the age of 40 and this situation becomes more prominent with the menopausal status in women. In the transplant population, the use of steroids and other immunosuppressive agents, additional comorbidities such as diabetes and decreased physical activity may cause the expected BMD decrease to be seen at an earlier stage and more severely [18]. Early menopause and decreased sex hormones in both genders frequently reported in the natural course of chronic kidney disease process, may also have an impact on the increased prevalence of LBD in relatively young transplant patients.

Serum alkaline phosphatase (ALP) is a marker of high turnover bone disease and we showed that there was a significant relation between ALP and LBD. Nonspecific total ALP is commonly used as a surrogate marker because of limited availability and high cost of bone specific ALP. Indeed, in one study a negative correlation was found between serum total ALP levels and BMD in hemodialysis patients [19]. In a transplant study published by Chandran et al., there was no significant correlation between total ALP levels and BMD but they found that BMD was significantly lower in patients with tertiary hyperparathyroidism [20]. In our study, the significant relationship between ALP levels and LBD may seem contradictory. We thought that the most important factor here could be the lower eGFR, especially in the osteoporosis group. The high turnover bone disease in low eGFR patients frequently results in high ALP levels. Nevertheless, there was no difference in PTH and 25-OH vitamin D as well as other bone markers such as osteocalcine and c-telopeptide between patients with and without LBD. Indeed, lack of any relationship between LBD and PTH, 25-OH vitamin D, osteocalcine and c-telopeptide were also reported by others [5, 9, 21, 22]. Our study suggests that the use of calcitriol and vitamin D supplements may disrupt the relationship between bone markers and BMD. High ALP level in patients with LBD indicates the importance of ALP follow-up in transplant patients.

Interestingly, we found that the risk factors for low bone density were quite different when analyzed separately regarding sex. In female recipients, menopausal status was the strongest risk factor for low bone density. However, in male recipients, mTOR inhibitor use and lower BMI level were the risk factors for LBD. Relationship between mTORi use and decreased BMD is a controversial issue. In-vitro studies demonstrate that sirolimus shows anti-proliferative effects in both osteoblasts and osteoclasts and it also helps final differentiation of osteoclasts [23–25]. Net results of these effects are increased bone turnover and bone mass loss. mTOR inhibitors can also slow the healing process after bone fracture [25]. Similar to our study, Gregorini et al. found that sirolimus use was the risk factor for osteoporosis development in KT recipients [10]. However, the specific negative effect of mTOR inhibitors on LBD in males has to be interpreted with caution and needs further investigation and validation in other studies.

In male recipients, BMI levels were significantly associated with LBD. In general population, epidemiologic data show that high body weight or high BMI is correlated with high bone density [26]. It has been generally accepted that increased mechanical burden associated with high BMI contributes to increase in bone density to accommodate the heavier body weight [27]. In our study this BMI effect on bone metabolism was seen only in males whereas it was not observed in female recipients. The relationship between BMI and bone density is thought to be mostly through total fat mass [28]. In the general population, higher the total fat mass means higher sex hormones in turn leading to better BMD. The reason why we could not reveal the relationship between BMI and bone density in women in our transplant cohort may be due to our female recipients’ decreased sex hormone reserve due to post-menopausal period and/or CKD [29]. This has also been reported in the general population [30].

Our study indicates that vertebral fractures of any grade have a very high prevalence (64.1%) even in our relatively young (mean age 46,5 years) KT recipients. The prevalence of ≥Grade 2 vertebral fractures was 43.4% in our KT population. This is a much higher prevalence than previously reported by others. Segaud et al. reported 11% vertebral fractures among kidney transplant recipient. Only lumbar vertebra examination and absence of detailed definition of the vertebral fractures were major pitfalls in this French cohort study [31]. In a Spanish cohort study, grade 2 or higher degree vertebral fracture prevalence was found in about 15% of all patients [9]. Besides geographic and genetic variations between different parts of the world, a multidisciplinary approach including an experienced endocrinologist with a special interest in osteoporosis in the team during the evaluation of the patients may account for our higher rate. The evaluation of the images by an experienced eye is essential not to miss the vertebral fractures. Furthermore the decision to initiate treatment for osteoporosis should be made again by an experienced endocrinologist.

When we examined the risk factors for vertebral fracture, unlike other studies we found no relationship in terms of age and gender. On the other hand, the prevalent vertebral fracture rate was significantly higher in patients with low AP lumbar vertebra BMD in our cohort.

Although it is well known that steroid induced bone loss is mainly driven by osteoclasts and osteoblasts, steroids also impair the function of osteocytes and stimulate their apoptosis resulting in impaired bone architecture, rendering the patients on steroid treatment could be more experienced more prone to fractures [32]. In our study, steroid use was not associated with LBD but prevalent fractures were more frequent in patients on steroids.

One of the options used to evaluate the fracture risk and eligibility for fracture prevention treatment is FRAX score. In the general population, FRAX accurately estimates the 10-year probability of MOF (e.g., composite of clinical vertebral, hip, forearm, and humerus) using an algorithm based on age, sex, clinical risk factors including previous fracture, parental hip fracture, presence of rheumatoid arthritis, smoking, secondary osteoporosis (organ transplantation), body mass index, prolonged steroid use, and alcohol intake with bone mineral density [2, 3, 14]. Although KT recipients may have a tendency to increased fracture risk compared to general population, the predictive value of FRAX in the KT recipients is unknown and there is a limited number of studies investigating FRAX in KT recipients [33, 34]. Naylor et al. reported the use of FRAX score in post-transplant patients and the observed 10-year MOF risk was 6.3%, which was in accordance with the predictions from the FRAX tool. Among their KT recipients, 1.5% of them had high MOF probability (≥20% risk of fracture), and 6.5% had high hip fracture probability (≥3% risk of hip fracture) and they conclude that FRAX showed modest MOF prediction with 0.62 value of area under curve and discrimination was similar to the general population [33]. In the Iranian study by Malakoutian et al., the number of the transplant patients with high hip fracture risk was reported as 11 among 70 (15.7%) [34]. However in this study, the authors did not examine fracture outcomes in relation to FRAX. In our cross-sectional study, we obtained comparable rates in high MOF risk (1.9%) but percentage of the patients with high hip fracture risk was high (23.5%). FRAX thresholds were created for women and men aged 50 and over. Therefore the major limitation of FRAX use in KT recipients is that transplant populations generally consist of relatively young patients. Besides genetic and geographic disparities, addition of CKD process and transplant specific clinical markers to FRAX calculation may improve the predictive capacity of FRAX. Longitudinal follow-up data would help us understand the value of FRAX in KT recipients.

The main limitations of our study are due to its retrospective design hence lack of bone-specific alkaline phosphatase levels, sex hormone profiles as well as bone histomorphometric data. Our study is also limited by the absence of densitometry data in the first year of transplantation, which prevented us from investigating loss of bone mass in the years following. However including only the patients who have completed at least one-year after transplantation provides homogeneity in our cohort of patients, since stabilization in bone density after the first year of transplantation is expected [20].

In conclusion, LBD in female KT recipients is mainly affected by menopausal status whereas in males, mTOR inhibitor use and lower BMI levels are the prominent risk factors. We can conclude that gender differences should be taken into account for bone evaluation in KT recipients. Further and longitudinal studies are needed to evaluate the value of FRAX in transplant population.

## Supporting information

S1 File (SAV)

S2 File (PDF)
