# Supplementary material for: Low bone density, vertebral fracture and FRAX score in kidney transplant recipients: A cross-sectional cohort study
Source: PLoS One. 2021 Apr 30;16(4):e0251035. doi: 10.1371/journal.pone.0251035 (PMC8087085; doi:10.1371/journal.pone.0251035)
Supplement: S2 File — (PDF) [file pone.0251035.s002.pdf]

Calculation Tool

Please answer the questions below to calculate the ten year probability of fracture with BMD.

Country: Turkey Name/ID:

Questionnaire:

1. Age (between 40 and 90 years) or Date of Birth
   Age: Date of Birth: Y: M: D:
2. Sex Male Female
3. Weight (kg)
4. Height (cm)
5. Previous Fracture No Yes
6. Parent Fractured Hip No Yes
7. Current Smoking No Yes
8. Glucocorticoids No Yes
9. Rheumatoid arthritis No Yes
10. Secondary osteoporosis No Yes
11. Alcohol 3 or more units/day No Yes
12. Femoral neck BMD (g/cm^2)
    Select BMD

Clear Calculate

Risk factors

For the clinical risk factors a yes or no response is asked for. If the field is left blank, then a "no" response is assumed. See also notes on risk factors.

The risk factors used are the following:

Age	The model accepts ages between 40 and 90 years. If ages below or above are entered, the programme will compute probabilities at 40 and 90 year, respectively.
Sex	Male or female. Enter as appropriate.
Weight	This should be entered in kg.
Height	This should be entered in cm.
Previous fracture	A previous fracture denotes more accurately a previous fracture in adult life occurring spontaneously, or a fracture arising from trauma which, in a healthy individual, would not have resulted in a fracture. Enter yes or no (see also notes on risk factors).
Parent fractured hip	This enquires for a history of hip fracture in the patient's mother or father. Enter yes or no.
Current smoking	Enter yes or no depending on whether the patient currently smokes tobacco (see also notes on risk factors).
Glucocorticoids	Enter yes if the patient is currently exposed to oral glucocorticoids or has been exposed to oral glucocorticoids for more than 3 months at a dose of prednisolone of 5mg daily or more (or equivalent doses of other glucocorticoids) (see also notes on risk factors).
Rheumatoid arthritis	Enter yes where the patient has a confirmed diagnosis of rheumatoid arthritis. Otherwise enter no (see also notes on risk factors).
Secondary osteoporosis	Enter yes if the patient has a disorder strongly associated with osteoporosis. These include type I (insulin dependent) diabetes, osteogenesis imperfecta in adults, untreated long-standing hyperthyroidism, hypogonadism or premature menopause (<45 years), chronic malnutrition, or malabsorption and chronic liver disease
Alcohol 3 or more units/day	Enter yes if the patient takes 3 or more units of alcohol daily. A unit of alcohol varies slightly in different countries from 8-10g of alcohol. This is equivalent to a standard glass of beer (285ml), a single measure of spirits (30ml), a medium-sized glass of wine (120ml), or 1 measure of an aperitif (60ml) (see also notes on risk factors).
Bone mineral density (BMD)	(BMD) Please select the make of DXA scanning equipment used and then enter the actual femoral neck BMD (in g/cm2). Alternatively, enter the T-score based on the NHANES III female reference data. In patients without a BMD test, the field should be left blank (see also notes on risk factors) (provided by Oregon Osteoporosis Center).

Notes on risk factors

Previous fracture

A special situation pertains to a prior history of vertebral fracture. A fracture detected as a radiographic observation alone (a morphometric vertebral fracture) counts as a previous fracture. A prior clinical vertebral fracture or a hip fracture is an especially strong risk factor. The probability of fracture computed may therefore be underestimated. Fracture probability is also underestimated with multiple fractures.

Smoking, alcohol, glucocorticoids

These risk factors appear to have a dose-dependent effect, i.e. the higher the exposure, the greater the risk. This is not taken into account and the computations assume average exposure. Clinical judgment should be used for low or high exposures.

Rheumatoid arthritis (RA)

RA is a risk factor for fracture. However, osteoarthritis is, if anything, protective. For this reason reliance should not be placed on a patient's report of 'arthritis' unless there is clinical or laboratory evidence to support the diagnosis.

Bone mineral density (BMD)

The site and reference technology is DXA at the femoral neck. T-scores are based on the NHANES reference values for women aged 20-29 years. The same absolute values are used in men.

Website Design Sheffield by Richlyn Systems
